# Neogene Proto-Caribbean porcupinefishes (Diodontidae)

**DOI:** 10.1371/journal.pone.0181670

**Published:** 2017-07-26

**Authors:** Orangel Aguilera, Guilherme Oliveira Andrade Silva, Ricardo Tadeu Lopes, Alessandra Silveira Machado, Thaís Maria dos Santos, Gabriela Marques, Thayse Bertucci, Thayanne Aguiar, Jorge Carrillo-Briceño, Felix Rodriguez, Carlos Jaramillo

**Affiliations:** 1 Universidade Federal Fluminense (UFF), Instituto de Biologia, Departamento de Biologia Marinha, e Programa de Pós-graduação em Biologia Marinha e Ambientes Costeiros, Niterói, RJ, Brasil; 2 Nuclear Instrumentation Laboratory, Nuclear Engineering Program/COPPE. Federal Univertsity of Rio de Janeiro (UFRJ), Rio de Janeiro, Brazil; 3 Palaeontological Institute and Museum, University of Zurich, Karl-Schmid-Strasse 4, Zürich, Switzerland; 4 Smithsonian Tropical Research Institute, Balboa, Republic of Panama; University of California, UNITED STATES

## Abstract

Fossil Diodontidae in Tropical America consist mostly of isolated and fused beak-like jawbones, and tooth plate batteries. These durophagous fishes are powerful shell-crushing predators on shallow water invertebrate faunas from Neogene tropical carbonate bottom, rocky reefs and surrounding flats. We use an ontogenetic series of high-resolution micro CT of fossil and extant species to recognize external and internal morphologic characters of jaws and tooth plate batteries. We compare similar sizes of jaws and/or tooth-plates from both extant and extinct species. Here, we describe three new fossil species including †*Chilomycterus exspectatus* n. sp. and †*Chilomycterus tyleri* n. sp. from the late Miocene Gatun Formation in Panama, and †*Diodon serratus* n. sp. from the middle Miocene Socorro Formation in Venezuela. Fossil Diodontidae review included specimens from the Neogene Basins of the Proto-Caribbean (Brazil: Pirabas Formation; Colombia: Jimol Formation, Panama: Gatun and Tuira formations; Venezuela: Socorro and Cantaure formations). *Diodon* is present in both the Atlantic and Pacific oceans, whereas the distribution of *Chilomycterus* is highly asymmetrical with only one species in the Pacific. It seems that *Diodon* was as abundant in the Caribbean/Western Atlantic during the Miocene as it is there today. We analyze the paleogeographic distribution of the porcupinefishes group in Tropical America, after the complete exhumation of the Panamanian isthmus during the Pliocene.

## Introduction

The uplift of the Central American isthmus [1,2,3] interrupted the Pacific-Atlantic seaway and drove large-scale rearrangement in the ocean circulation [4,5,6,7]. It produced environmental changes that distinguish today’s Eastern Pacific and Western Atlantic habitats. The Pacific side is characterized by productive surface water caused by coastal upwelling and abundant fast-growing suspension and detritus feeders on the sea bottom. By contrast, the Caribbean is characterized by more carbonate-rich habitats, nutrient-poor waters, and abundant seagrass and large coral reef assemblages in shallow waters [8,9]. There are also large Miocene-Pliocene hydrographic changes along the northern border of South America, including the Paleo Amazon-Magdalena-Orinoco fluvial system, with complex river delta systems producing high freshwater discharge to the South Caribbean and extensive estuarine environments [10,11,12,13]. These early hydrographic system could be linked with the origins of marine-derived freshwater fishes, including extant pufferfishes species (Tetraodontiformes) [14].

Current discussions about the uplift of the Central American isthmus suggest a gradual emergence of the volcanic Panama Arch [15], which started with the formation of a land bridge connecting central Panama with North America during the Aquitanian (early Miocene) [16], a subsequent and progressive formation of the isthmus during the Langhian/Serravallian (middle Miocene) [2], and a final closure of the Pacific-Atlantic connection by the complete uplift of the Isthmus of Panama during the early Pliocene [1,2,3,17].

The formation of the Isthmus of Panama should not be seen as an isolated but global event integrating data at the regional and global levels as complex Atlantic-Pacific teleconnections, such as currents, winds, salinity and temperature of waters, as well as paleodepth and paleoenvironments of deposition of the geological units [9, 18,19].

The history of Central Western Atlantic and Central Eastern Pacific distribution of fossil fish records during the Neogene has been closely related to the evolution of the oceanic pathway connecting both oceans across the Panamanian isthmus [20,21,22,23].

Extant porcupinefishes (Diodontidae) comprise seven genera and 18 species [24,25]. *Diodon* and *Chilomycterus* are today widely distributed in all circumtropical regions [24]. *Diodon*, the most common and widespread porcupinefish, is represented by five species: *Diodon eydouxii*, *D*. *holocanthus D*. *hystrix*, *D*. *nicthemerus* and *D*. *liturosus* [24,26]. *Chilomycterus* also is represented by five species: *Chilomycterus antennatus*, *C*. *antillarum*, *C*. *reticulatus*, *C*. *schoepfii* and *C*. *spinosus* [24,26]. In the oceanic regions of the Americas, there are three species of *Diodon*, along both the Eastern Pacific and Western Atlantic coasts; in contrast, from the Eastern Pacific *Chilomycterus*, is represented by one species [26]. Genetic studies of extant populations of Diodontidae have revealed that the divergence times of sister species do not extend beyond the early Oligocene [27]. The oldest record of Diodontidae in South America comes from the Cretaceous of Brazil [28].

Neogene Diodontidae from Tropical America were previously recorded in shallow water deposits from the early Miocene of Brazil [29], early Miocene of Venezuela [30], Miocene of Panama [31,32], middle-late Miocene to early Pliocene of Trinidad [33], middle Miocene of Florida (USA) [34], late Miocene of Cuba [35], and Pleistocene of Jamaica [36]. Along the Atlantic coast of the USA, these fishes also have been found in the Pliocene of North Carolina [37,38]. Fossil skeletons of Diodontidae from the Neogene of Tropical America have been poorly represented and consist mostly of isolated and fragmentary jawbones. The massive and fused beak-like jawbones characterize these fishes as powerful shell-crushing predators on tropical coral and rocky reefs and surrounding flats [39,40]. These durophagous fishes exploit environments with diverse and abundant shallow-water invertebrate faunas, mostly mollusks, echinoids and crustaceans [41,42,43,44].

Morphological [39,45,46,47,48] and molecular studies support the monophyly of Diodontidae [49,50,51], with *Chilomycterus* being sister to *Diodon*. Their divergence time (node-base age of all molecular based dates) has been estimated variously at 10.9 Ma [52], 18 Ma [49], 20.8 Ma [51], and 55.6 Ma [53].

Here, we review and describe three new fossil Diodontidae species from the Neogene Basins of the Proto-Caribbean, and analyze the paleogeographic distribution of the group in Tropical America, after the complete exhumation of the Panamanian isthmus during the Pliocene.

## Materials and methods

**Fig 1:**
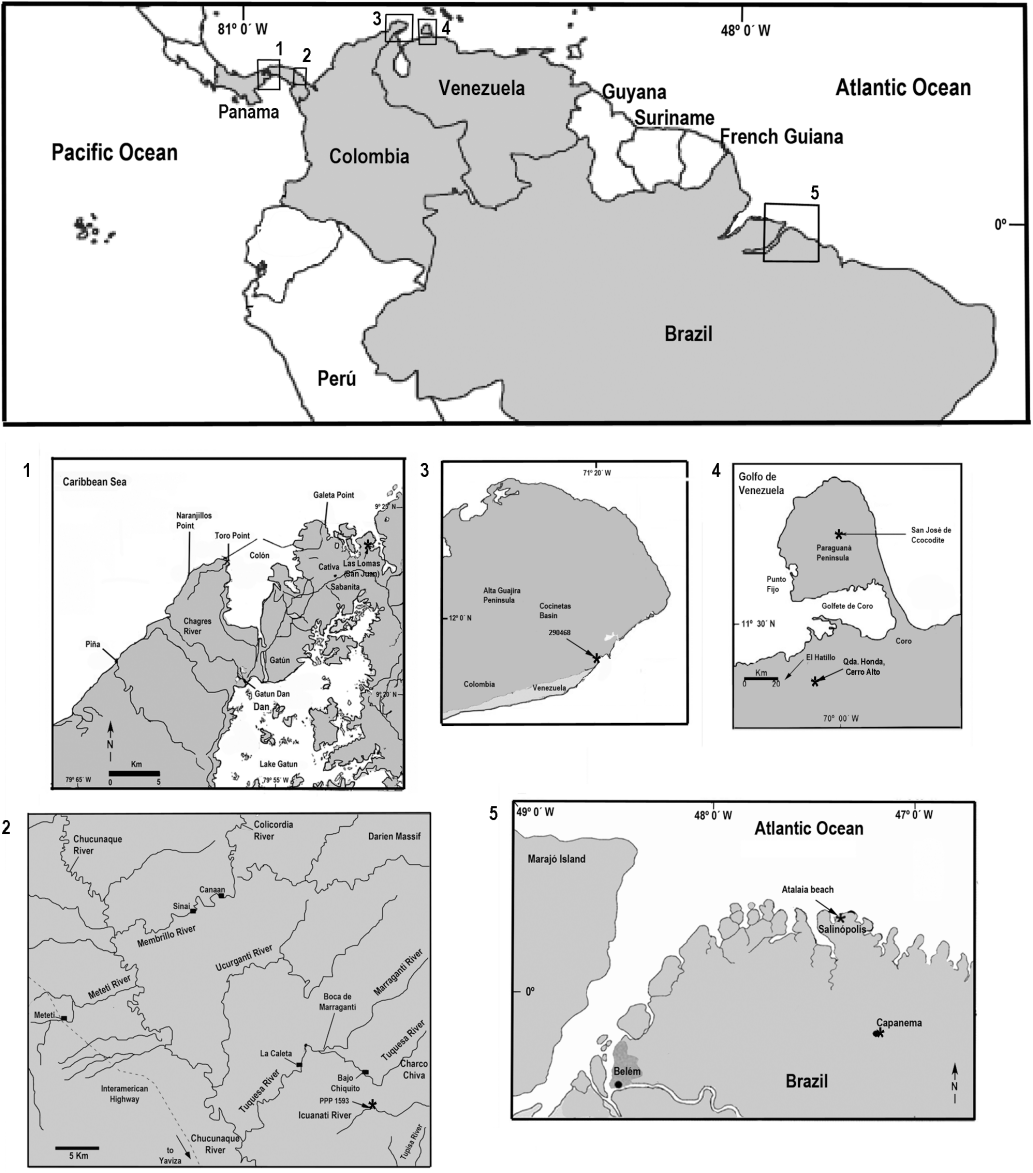
Locations maps. Panama (1, 2), Colombia (3), Venezuela (4) and Brazil (5).

**Fig 2:**
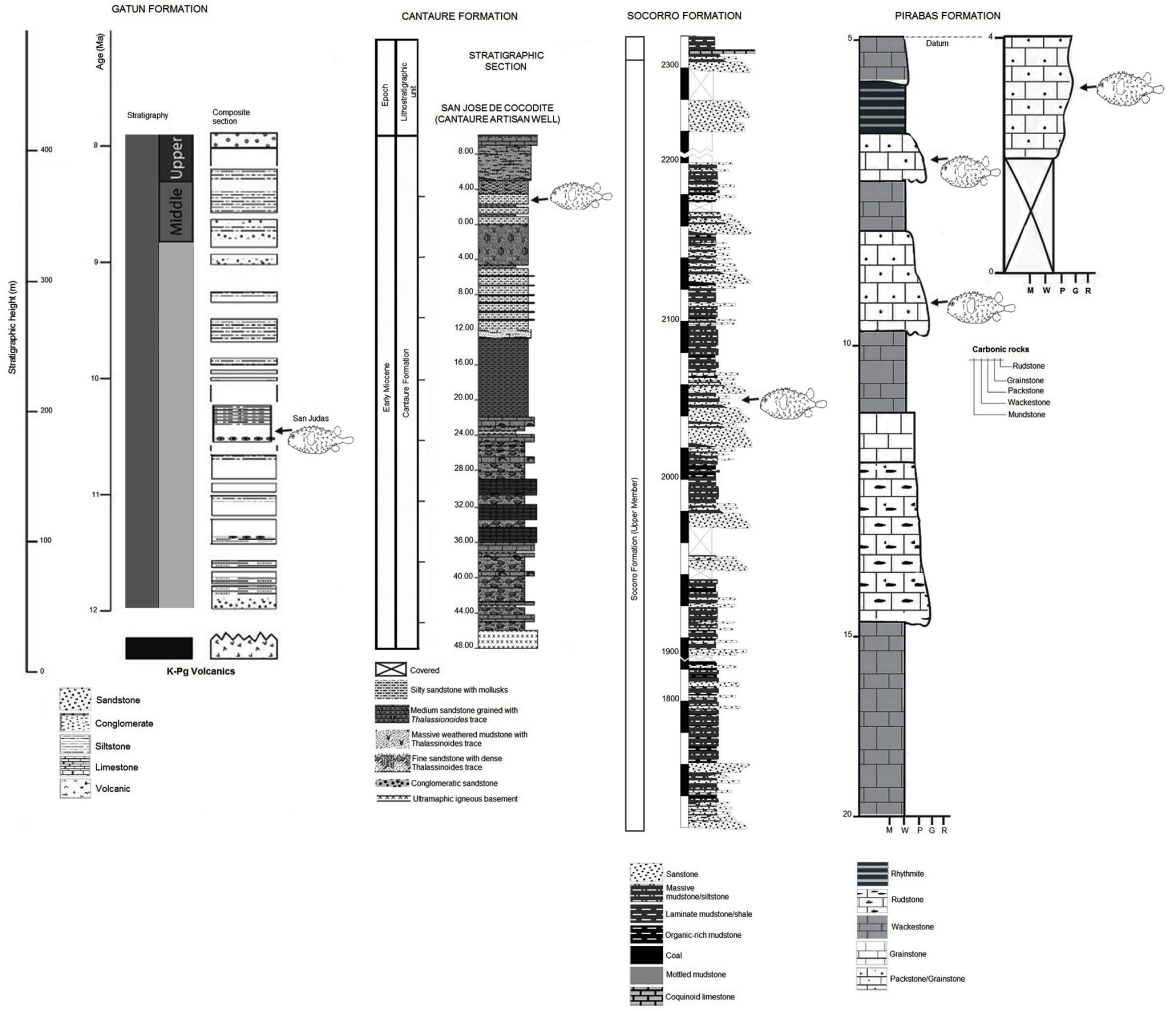
Stratigraphic sections and Diodontidae occurrence. Gatun Formation of Panama [54], Cantaure Formation of Venezuela [55], Socorro Formation of Venezuela [56], Pirabas Formation of Brazil [57]. Sections that not specifically related to fossil diodontids were erected from the Tuira (Panama) and Jimol (Colombia) formations.

**Fig 3:**
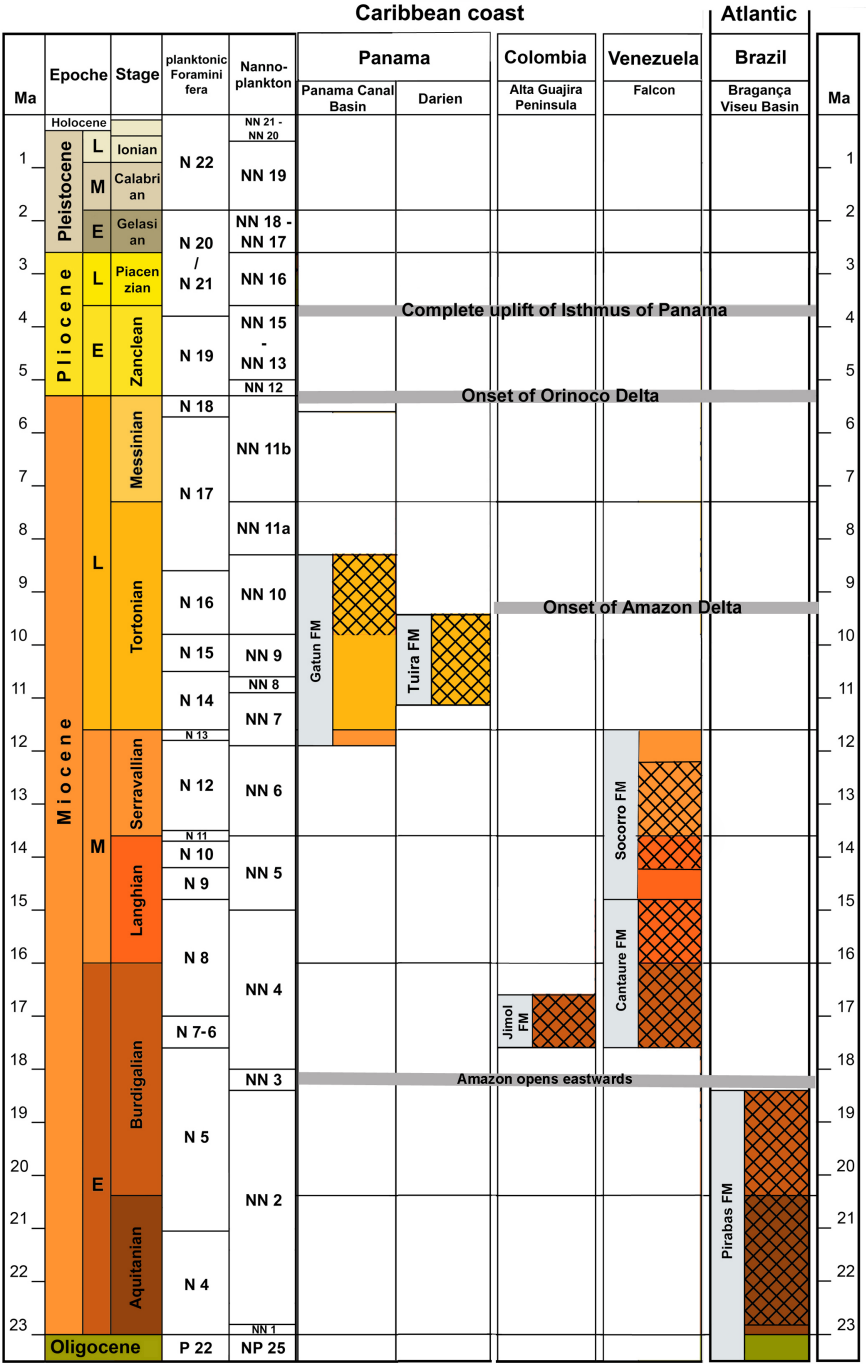
Correlations of the Neogene formations of tropical America with fossil Diodontidae treated herein [21]. Major events after Jaramillo [58,59].

**Fig 4:**
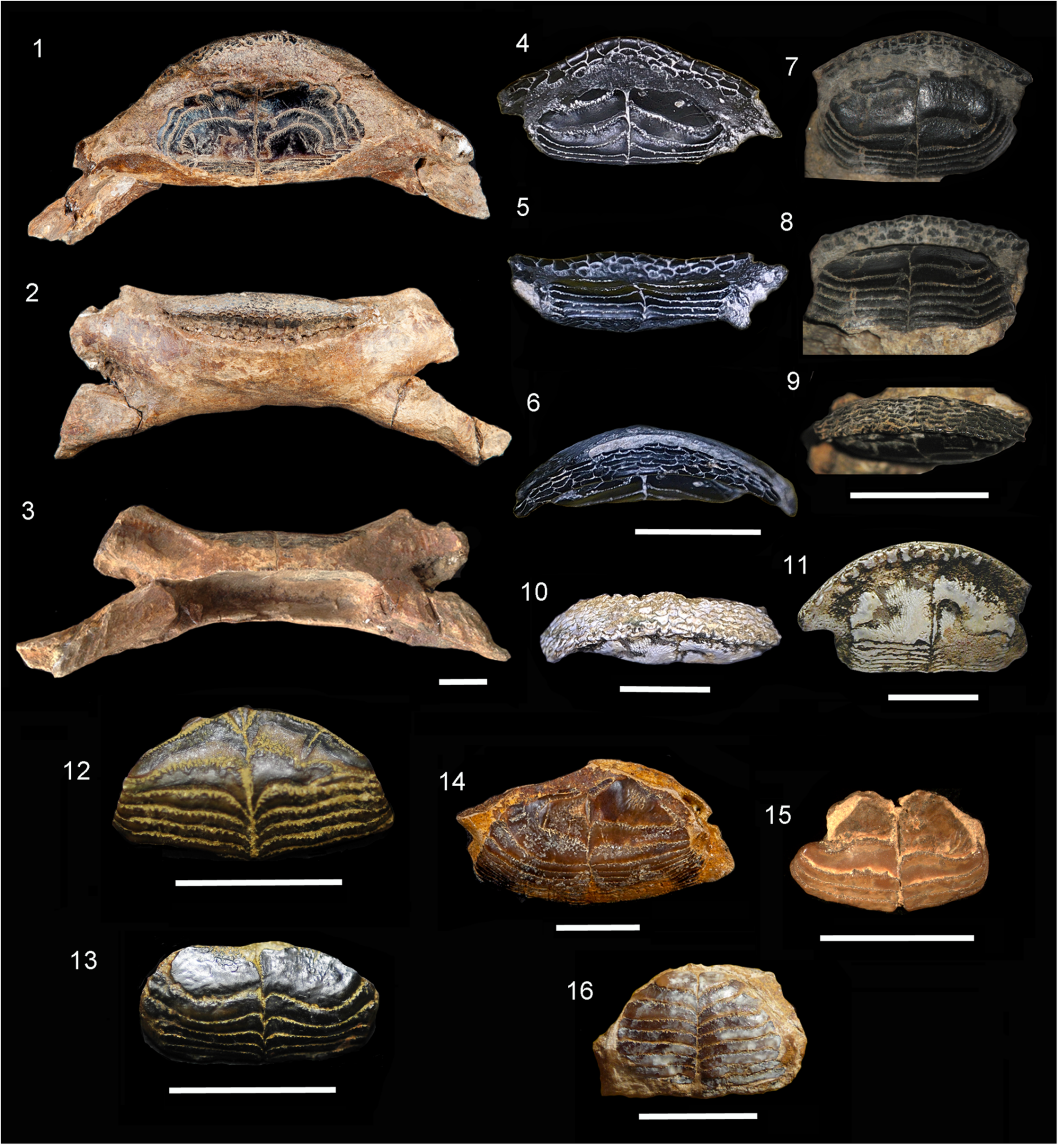
Fossil Diodontidae. **1–3**. †*Chilomycterus tyleri* n. sp., lower jaw, 99.8 mm in width, late Miocene Gatun Formation, Las Lomas, San Judas Tadeo, Colón, Panama, holotype, NMB P1208 (1, occlusal; 2, anterior; 3, posterior views). **4–6**. †*Chilomycterus ferreirai* (Santos and Travassos 1960) [29], upper jaw, 24 mm in width, early Miocene Pirabas Formation, Praia de Atalaia, Salinópolis, Brazil, MPEG 2084-V (4, occlusal; 5, posterior; 6, anterior views). **7–9**. †*C*. *ferreirai*, lower jaw, 16.2 mm in width, early Miocene Pirabas Formation, Praia do Castelo, Ilha de Fortaleza, São João de Pirabas, Brazil, holotype, MN 2649-V (7, occlusal; 8, posterior; 9, anterior views). **10–11**. †*C*. *ferreirai*, upper jaw, 25.0 mm in width, early Miocene Cantaure Formation, San José de Cocodite, Venezuela, UNEFM-PF-270 (10, anterior; 11, occlusal views). **12**. †*C*. *exspectatus* n. sp., upper tooth plate battery, 20.2 mm in width, late Miocene Gatun Formation, San Judas Tadeo, Colón, Panama, holotype, MNB P1205 (occlusal view). **13**. †*C*. *exspectatus* n. sp., lower tooth plate battery, 16.64 mm in width, late Miocene Gatun Formation, San Judas Tadeo, Colón, Panama, paratype, MNB P1206 (occlusal view). **14**. *Chilomycterus* sp. tooth plate battery, 28.8 mm in width, middle Miocene Tuira Formation, Rio Icuanati, small tributary from village Boca de Marraganti (loc. PPP 1593), Darien, Panama, MNB P1207 (occlusal view). **15**. *Chilomycterus* sp., tooth plate battery, 13.0 mm in width, Jimol Formation, late early Miocene, Guajira Peninsula, Colombia, MUN-STRI- 41506 (occlusal view). **16**. †*Diodon serratus* n. sp., tooth plate battery, 18.0 mm in width, middle Miocene Socorro Formation, Quebrada Honda, Urumaco, Venezuela, holotype, AMU-CURS-760 (occlusal view). Scale bar 10 mm.

**Fig 5:**
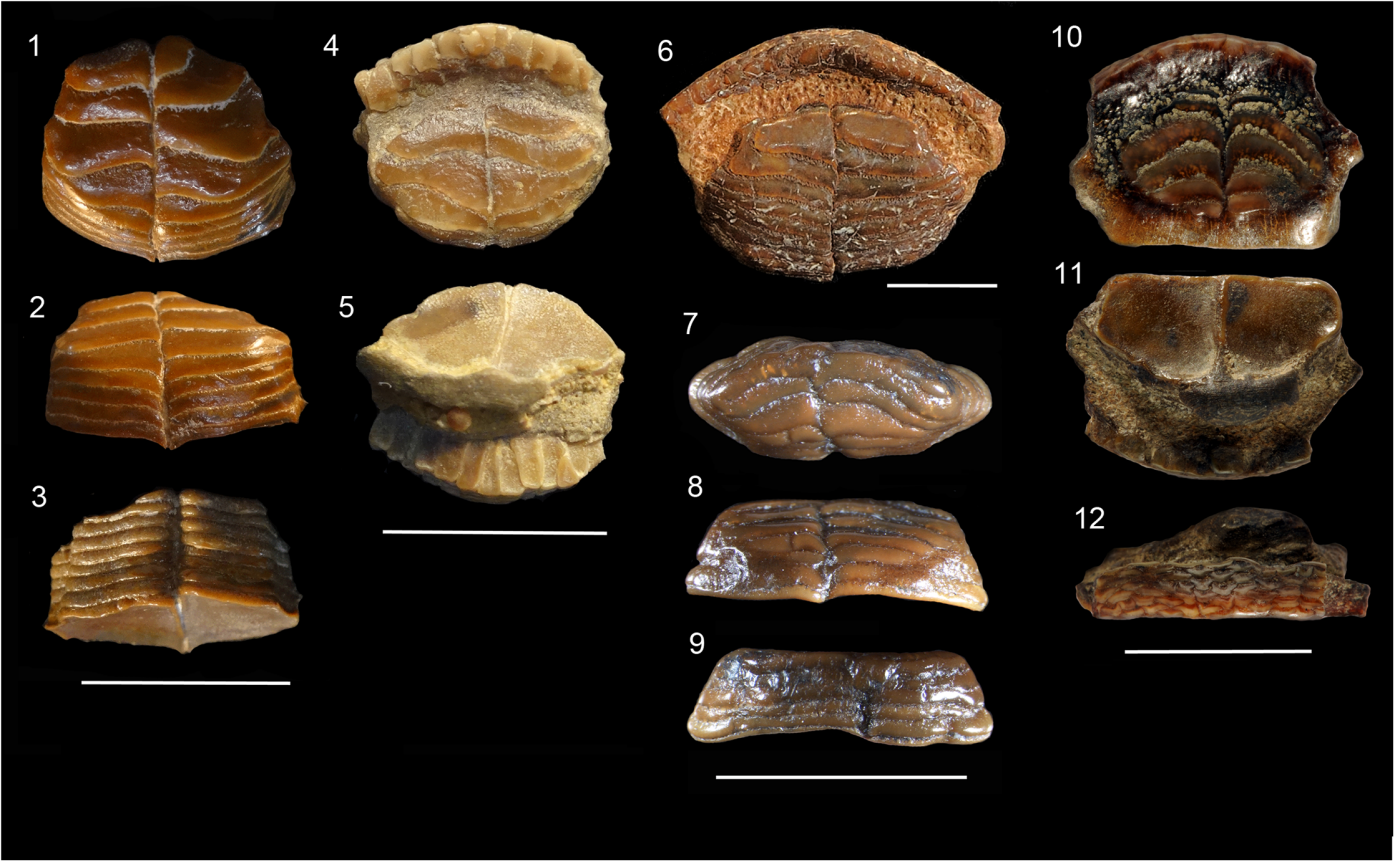
Fossil Diodontidae. **1–3**. †*Chilomycterus kugleri* (Casier 1958) [33], holotype, tooth plate battery, late Miocene, Gross Morne Formation, Trinidad, NMB-Ant.58 (1, occlusal; 2, anterior; 3, posterior views). **4–5**. †*C*. *vetus* (Leidy 1877) [37], upper jaw, middle Miocene Tamana Formation, Trinidad. NMB-Ant.57 (4, occlusal; 5, antero-dorsal views). **6.** †*C*. *circunflexus* (Leriche 1942) [34], upper jaw, provably late Miocene, La Cueva Sin Nombre, La Havana, MNHNH-P2083, occlusal view; **7–8**. †*C*. *circunflexus*, lower tooth plate battery, middle Miocene, Caspersen Beach 2, Venice, Florida, USA, PIMUZ A/I 3651. (7, occlusal; 8, anterior; 9, posterior views). **10–12**. †*C*. *gatunensis* (Toula, 1909) [31], holotype, upper jaw, late Miocene Gatun Formation, Panama, NHMW 1933/XVIII/167. (10, occlusal; 11, antero-dorsal; 12, anterior views). Scale bar 10 mm.

**Fig 6:**
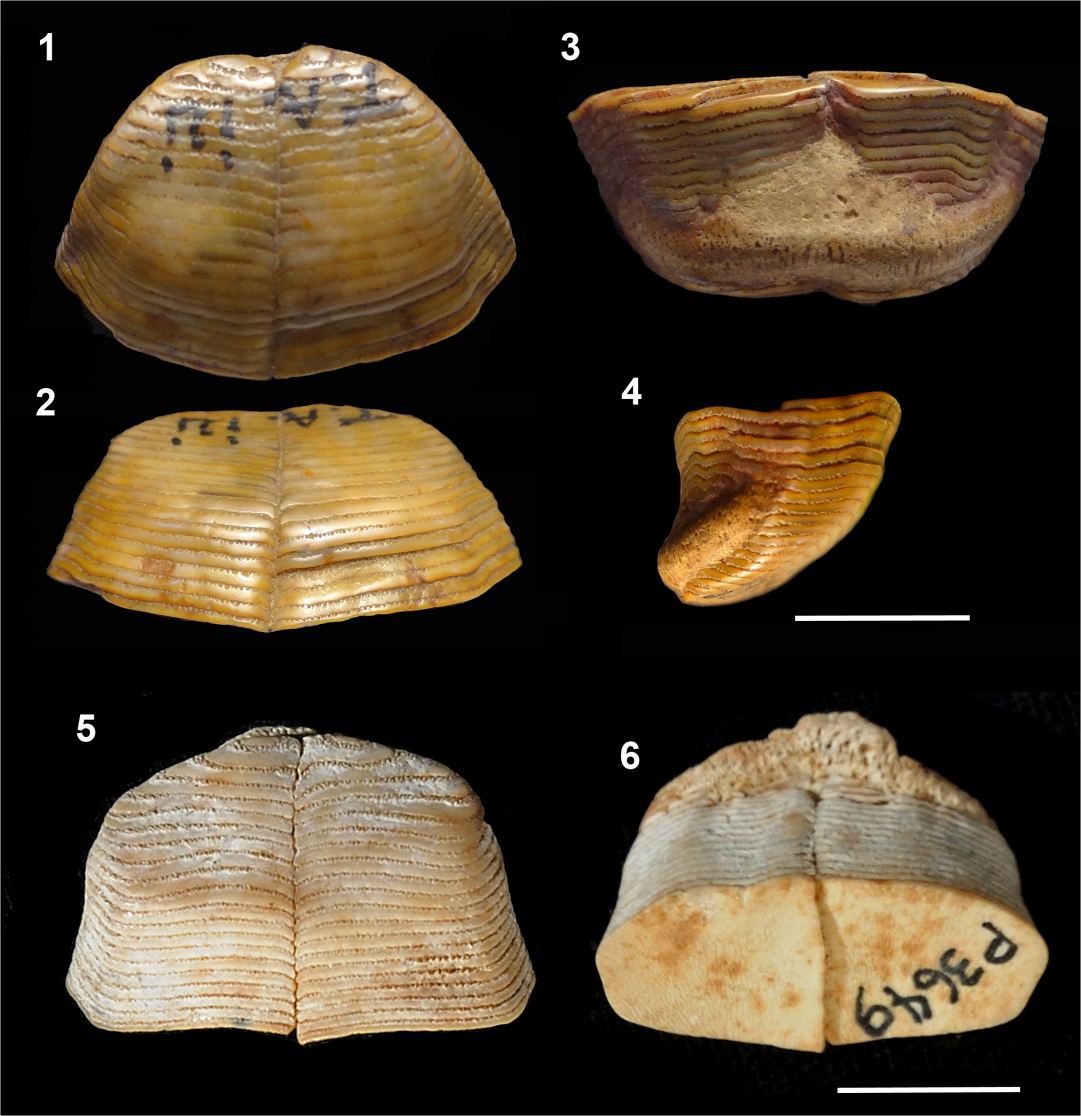
Fossil Diodontidae. **1–4**. †*Diodon sigma* Martin 1883 [60], tooth plate battery, Miocene, Java Island, Indonesian, MNB-T.A.121. (1, occlusal; 2, anterior; 3, posterior; 4, lateral views). **5–6**. †*D*. *scillae* Agassiz 1843 [61], tooth plate battery, Cueva sin Nombre, La Havana, Cuba, MNHNH- P3646. (5, occlusal; 6, basal views). Scale bar 10 mm.

**Fig 7:**
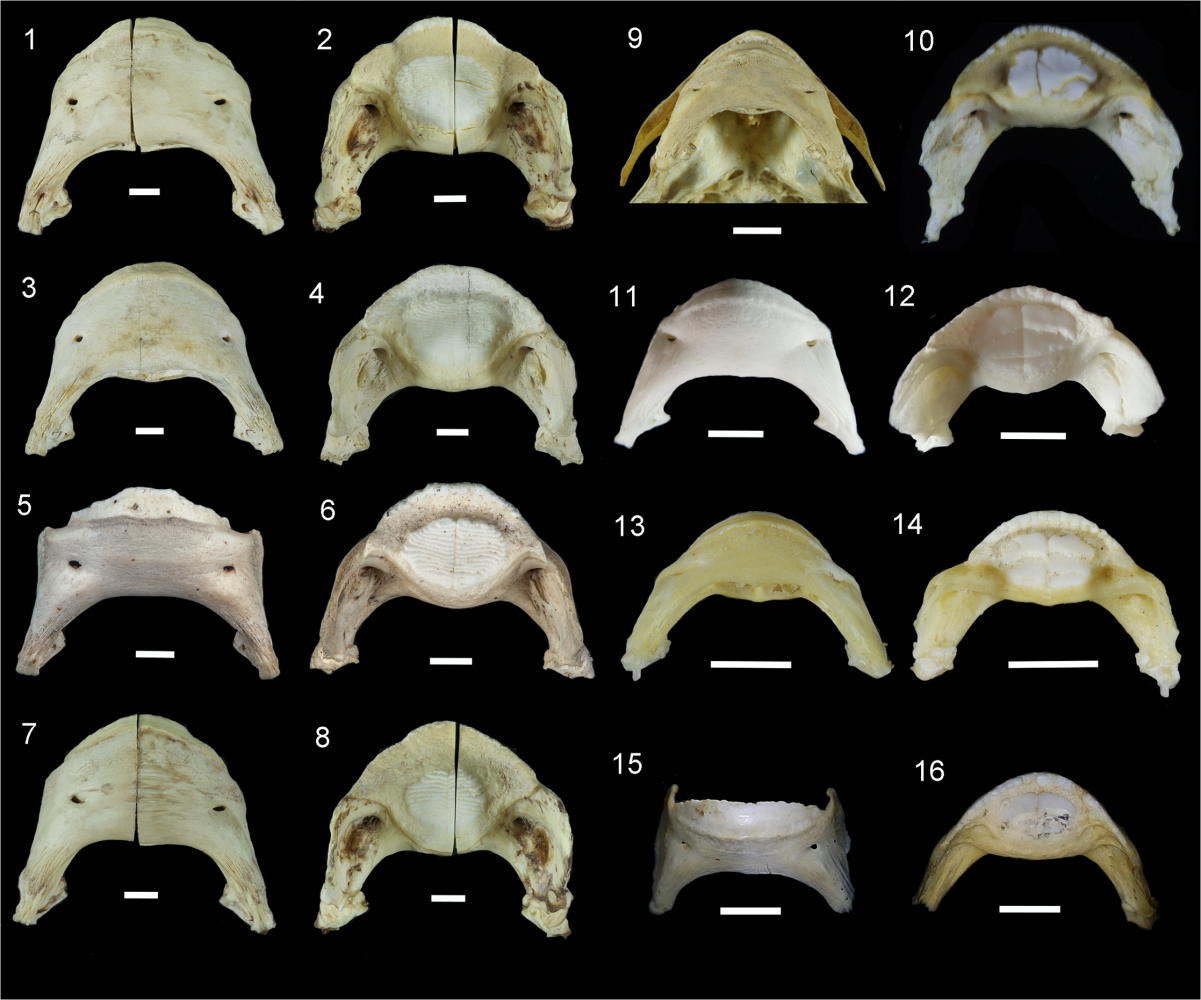
Extant Diodontidae. **1–2.** *Diodon hystrix* Linnaeus 1758 [62], lower jaw, Indian Ocean, Seychelles Islands, ANSP 102789, 505.0 mm standard length specimen (SL). (1, external; 2, occlusal views); **3–4.** *D*. *hystrix*, lower jaw, Caribbean, Puerto Rico, ANSP 109515, unavailable SL. (3, external; 4, occlusal views); **5–6**. *D*. *hystrix*, lower jaw, Caribbean, Venezuela, UFF ZO426, unavailable SL. (5, external; 6, occlusal views); **7–8.** *D*. *holocanthus* Linnaeus 1758 [62], lower jaw, Indian Ocean, Seychelles Islands, ANSP 102787, 375.0 mm SL specimen. (7, external; 8, occlusal views); **9.** *D*. *liturosus* Shaw 1804 [63], lower jaw, Indian Ocean, India, ANSP 109145, unavailable SL. (9, external view); **10.** *Chilomycterus nicthemerus* (Cuvier 1818) [64], articulate lower jaw, Port Phillip, Bass Straight and vicinity, Australia, AMNH 219858, unavailable SL. (10, occlusal view); **11–12.** *C*. *antillarum* Jordan and Rutter 1897 [65], lower jaw, Caribbean, Guadeloupe, MNHN 971–9506.0023, unavailable SL. (11, external; 12, occlusal views); **13–14.** *C*. *schoepfii* (Walbaum 1792) [66], lower jaw, Gulf of Mexico, Florida, USA, ANSP 109514, unavailable SL. (13, external; 14, occlusal views); **15–16.** *C*. *spinosus* (Linnaeus 1758) [62], lower jaw, Western Atlantic, Itaipú, Brazil, UFF ZO132, 184 mm SL specimen. (15, anterior; 16, occlusal views). (photos ANSP specimens by K. Luckenbill; AMNH R. by Arrindell; UNEFM by A. Bertoncini; MNHN by M. Lopes).

**Fig 8:**
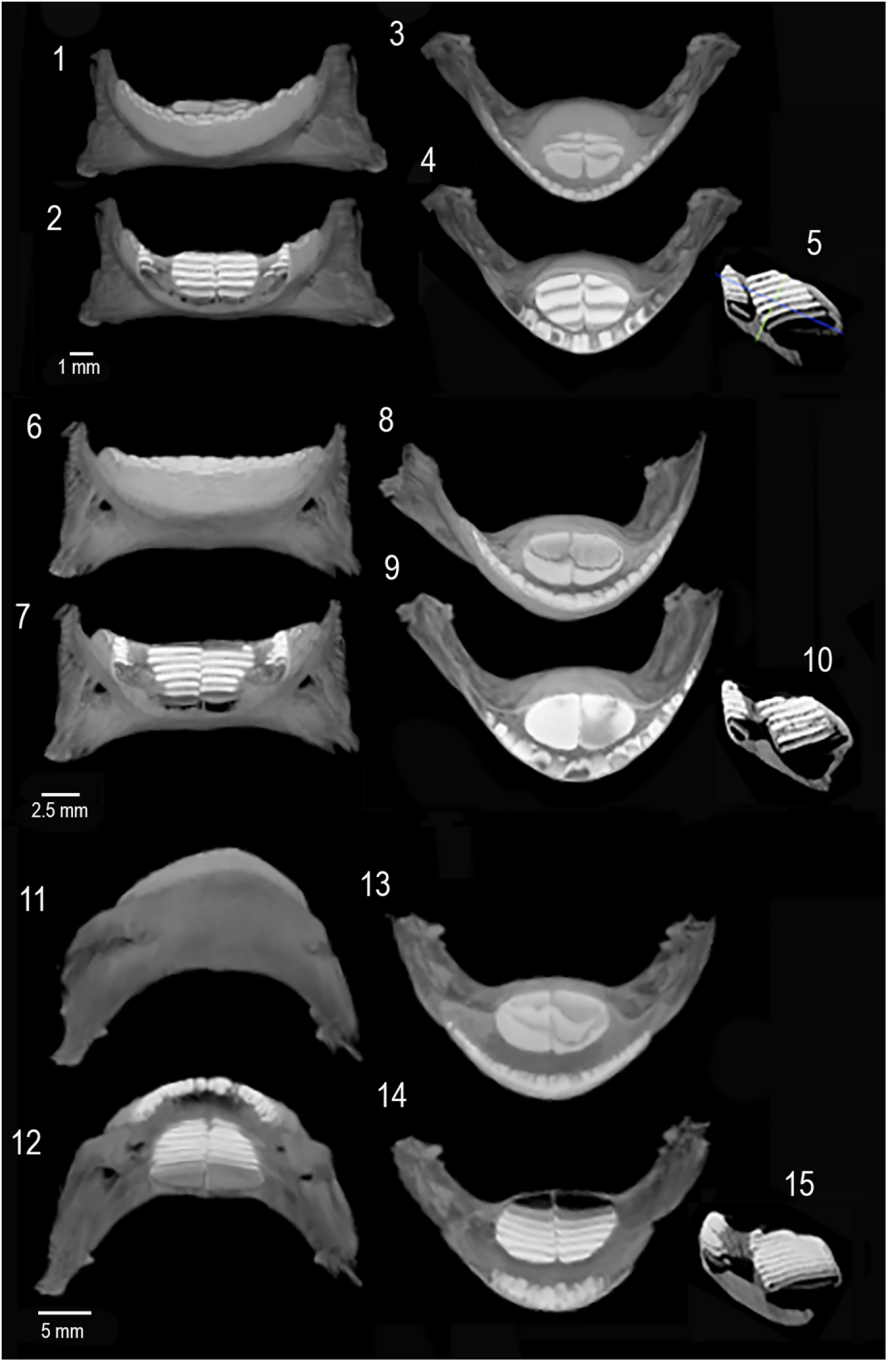
Micro CT plates of ontogenetic series of jaws from extant *Chilomycterus spinosus* from Santos, São Paulo, Brazil. 1–5, UFF ZO314: 92 mm total body length (TL), 72 mm standard length (SL); 6–10,UFF ZO312: 159 mm TL, 120 mm SL; 11–15, UFF ZO315: 276 mm TL, 230 mm SL.

Fossils consist of isolated jaws and tooth plate batteries collected from six Miocene localities from the Pirabas (Brazil), Jimol (Colombia), Gatun and Tuira (Panama), and Socorro and Cantaure (Venezuela) formations (Figs 1, 2 and 3). Specimens were collected from outcrops during several independent expeditions by O. Aguilera, F. Rodriguez, C. Jaramillo and J. Carrillo-Briceño. The specimens are housed in the paleontological collections of the Museu Paraense Emilio Goeldi, Belem (MPEG); Museu Nacional do Rio de Janeiro (MN-UFRJ); Departamento de Geologia, Universidade Federal do Rio de Janeiro (DG-UFRJ), Brazil; Mapuka Museum of Universidad del Norte (MUN), Barranquilla, Colombia; Naturhistorisches Museum of Basel (NMB), Switzerland; Paleontological collections of the Alcaldía del Municipio de Urumaco (AMU-CURS) and Universidad Experimental Francisco de Miranda (UNEFM), Venezuela. Taxonomic identification of examined species (Fig 4, S1 File), comparative studies of fossil (Figs 5 and 6, S2 File) and extant species (Figs 7 and 8, S3–S7 Files) were based on the ichthyological collections of the Academy of Natural Sciences of Philadelphia (ANSP), USA; American Museum of Natural History (AMNH), USA; Museum of Natural History of Wien (NHMW), Austria; Muséum National d'Histoire Naturelle (MNHN), France; Naturhistorisches Museum of Basel (NMB), Switzerland; Museo Nacional de Historia Natural de La Havana (MNHNH), Cuba; Universidade Federal Fluminense (UFF), Brazil, and on extensive bibliographical review. We gathered habitat information of living representatives from the FishBase website [67] and additional updated records [68,69]. The maps of geographical distribution were based on IPCC A2 emissions scenario [www.aquamaps.org, version of Aug. 2013].

3D high-resolution micro CT scans of fossil and recent lower-jaw specimens were obtained from a desktop system (Bruker/Skyscan 1173), which was calibrated to operate at 55 kV voltage and 145μA current with a flat-panel detector matrix of 2240 x 2240 pixels. A pixel size of 14.8 μm was used with a scanning step rotation along the *z* axis of 0.8° over 360°. The longest axis (thicker part) of each sample was aligned perpendicular to the rotation axis of the equipment, which provides the shortest X-ray path length through the material. This is important because it results in different degrees of beam hardening effect. Sets of these micro CT projections were reconstructed by using a mathematical process called filtered back projection [70]. By reducing the artifacts produced by beam hardening and ring, which are inherent to the scanning [71], we were able to improve the image quality.

### Geological context and locations

The fossil jaws and tooth plate batteries of Diodontidae were obtained from a variety of Neogene formations from Tropical America (Fig 1). Fig 2 summarizes the stratigraphic context of the fossil collections.

#### Brazil

The Pirabas Formation [72] is upper Oligocene to lower Miocene and consists of carbonate coquinas accumulated in an offshore platform environment (grainstone and consolidated packstone, stratified wackestone to packstone and laminated mudstone). In addition, littoral facies (shoreface/foreshore), marginal lagoons, restricted platform environments (grey to olive mudstone and conglomeratic sandstone) and mangrove estuarine lagoons (dark mudstone, massif or laminated) have been recorded [55,73,74,75,76,77]. Planktonic foraminifera associations [78,79,80,81,82] suggest an early Miocene age, Aquitanian to early Burdigalian, N4 to N5 plankton foraminiferal biozones [83]. A sample from the Pirabas Formation yielded a rich and well-preserved palynological assemblage dominated by terrestrial organic matter and the taxa *Echiperiporites estelae* (Malvaceae), *Retitrescolpites*? *irregularis* (Phyllanthaceae), *Rhoipites guianensis* (Malvaceae), *Lanagiopollis crassa* (Pellicieraceae), *Perfotricolpites digitatus* (Convolvulaceae), *Cricotriporites macroporus* (angiosperm), *Mauritiidites franciscoi var*. *franciscoi* (Araceae), *Monoporopollenites annulatus* (Poaceae), *Echinatisporis muelleri* (fern), *Magnastriatites grandiosus* (Ceratopteris), as well as some dinoflagellates (*Achomosphaera* sp.). An early Oligocene to early Miocene age can be deduced from the co-occurrence of *Lanagiopollis crassa*, *Rhoipites guianensis*, *Cricotriporites macroporus*, *Echinatisporis muelleri* and *Magnastriatites grandiosus* [84,85,86]. A diverse fossil fauna has been described, rich in mollusks, decapods and fishes [60,75,80]. Locations (Fig 1.5): Praia de Atalaia, Salinópolis Municipality, Pará state, Brazil (Fig 5) [57].

#### Colombia

The Jimol Formation [87] is composed of grey calcareous sandstone, yellowish-grey biosparites, and grey to brown siltstones and mudstone. At the base occur 50 cm to 1 m thick beds of coarse calcareous sandstone with ripples, cross and planar bedding. Wackestone to packstone biosparites dominate the sequence. There are occasional ~5 m thick beds of siltstone and mudstone in this part of the sequence. At the top mudstone and fine-grained calcareous sandstone in 5 m to 20 m thick beds dominate the sequence, interbedded with 50 cm to 2 m thick beds of fine to medium-grained calcareous sandstone, and wackestone to packstone biosparites. A late early Miocene (Burdigalian) age is assigned to the Jimol Formation on the basis of macro-invertebrate biostratigraphy and ^87^Sr/^86^Sr isotope chronostratigraphy [88]. A diverse fossil mollusk fauna from the marginal marine shallow waters of the Jimol Formation has been described [89]. Locations (Fig 1.3): locality 290468, in Padsua Sur, early Miocene Jimol Formation (early Burdigalian), La Guajira Peninsula, Colombia (Fig 2) [88,90].

#### Panama

The Gatun Formation [91] is divided into three members [92]. A section of the lower member, dated as 11.7 to 9 Ma (late Miocene) [54], is exposed along the trans-isthmian highway about 12 km east of Colón City, from Sabanita to Cativa. This member consists of burrowed, concretionary, grey-green, tuffaceous, and silty litharenite, which is interpreted as representing a nearshore (paleo-depth ~11 to ~ 65 m), sandy and soft-bottom environment [54,93]. This section has yielded the diodontid jawbones described herein. The total thickness of the Gatun Formation, recorded in a borehole near Colón City, is about 500 m [92]. Locations (Fig 1.1): ID 42501, Las Lomas, San Judas Tadeo, lower Gatun Formation, Panama (Fig 2) [54].

The Tuira Formation [94,95] from the Chucunaque-Tuira basin consists of thin and regularly bedded alternations of blue gray greywacke and arkosic sandstone with dark green to black, silty claystone and siltstone. Abundant plant debris, scattered small mollusks, particularly pectinids and nuculanids are present. Many units have pervasive bioturbation or thalassinoid burrow systems. Pebble breccia, shell beds, and stringers of rip-up clasts may occur occasionally. The upper part of the Tuira Formation ranging between 11.2 to 9.4 Ma (late Miocene) [95]. The tooth plate battery has been collected from the upper part. Location (Fig 1.2): PPP 1593, small tributary of Río Icuanati, Darien.

#### Venezuela

The Cantaure Formation [96] is of late early Miocene, in age, late Burdigalian to early Langhian (NN4-5, N7-8). Its stratotype is located approximately 10 km west of Pueblo Nuevo on the Paraguaná Peninsula, Falcón state, Venezuela. Outcrops of the formation are found west of Casa Cantaure and are composed of silty shales interbedded with thin algal limestones and shell beds [96]. An unexposed unit of Cantaure Formation, 48 m thick, was accessed by a local artesian well. The section consists mainly of silty to medium grained sandstone, intercalated with massive mudstone. Planktonic foraminifera and calcareous nannofossils revealed a late Burdigalian to early Langhian age [97,98]. A diverse fossil fauna has been described, rich in mollusks, decapods, fishes and mammals [30,99,100,101,102,103,104,105]. The fossil composition is indicative of a tropical-marine, clear-water near shore neritic environment of normal marine salinity, probably not far from open marine environments [97,99,101,104,106]. The base of the Cantaure Formation is dated at about 16.5 Ma [104]. Location (Fig 1.4): San José de Cocodite, pozo Cantaure, Paraguaná Peninsula (Fig 2B) [55], (Fig 1) [104].

The Socorro Formation [107] is middle Miocene in age. The section, located along Paují Creek; 20 km east of the town of Urumaco, the unit is 2300 m thick and it has been divided into three members (lower, middle and upper) [56]. The specimen referred here was collected in a coquinoid layer of the upper member together with a few otoliths as well shark and rays teeth [13]. Location (Fig 1.4), Cerro Alto, Quebrada Honda.

### Differential morphology of tooth-plates in *Diodon* and *Chilomycterus*

Osteological research in extant Diodontidae conducted by Tyler [45] revealed an exponential increase in the number of dental sheets in the tooth batteries of *Diodon* (~5 to 40) as a function of the increase in the standard length (63–550 mm). Therefore, in the ontogenetic series (S1 Fig) the early stages in *Diodon* (specimen size <220 mm) overlap all *Chilomycterus* specimens with standard length of 72 to 580 mm): 5–20 dental sheets in *Diodon* vs. 7–18 in *Chilomycterus*. However, unlike the large body-size specimens of *Diodon*, which have more than 35 dental sheets, a large specimen of *C*. *reticulatus*, with standard length ~580 mm has only 18 dental sheets. The ontogenetic series of *Diodon* jaws reveals that the frontal teeth form a cutting edge distant from the crushing tooth-plate batteries. Therefore, the massive tooth-plate batteries are restricted to the posterior most area of the occlusal surface of the mandible. In contrast, the cutting edge of the jaw in *Chilomycterus* is close to the tooth-plate batteries; it begins with the front tooth series, surrounds the grinding surface, and continues posteriorly along the jaw.

These durophagous morpho-functional differences are characterized by massive and high tooth-plate batteries in *Diodon* vs. slender and low tooth-plate batteries in *Chilomycterus*. In *Diodon*, the ontogenetic changes in the arrangements of the crushing surface could be associated with food preference; mollusks (44.5 to 70.5%), echinoids (11.6 to 34.6%) and crustaceans (20.8 to 37%) are the most important food [41]. Large *Diodon hystrix* individuals are able to crush exceptionally strong shells, exerting a force of 5000 N (equivalent of 500 Kg load) [44,108]. In contrast, specimens of *Chilomyctetrus* (*C*. *schoepfi*) with around 20 cm body size are capable of generating a force of only 380 N (equivalent of 38 Kg load) in the crushing plate [109]. *Chilomycterus* food preferences consist mostly of mollusks (56.6%) and crustaceans (43.4%) [41].

Fossil species of Diodontidae from the Neogene described by Schultz [110] had ~37 dental sheets in the tooth battery in *Diodon* and 8–12 in *Chilomycterus*. We use an ontogenetic series of micro CT scans to quantify and compare the number of dental sheets in the tooth-plate batteries (S2–S7 Files). We compare similar sizes of jawbones and/or tooth-plates from both extant and extinct species of *Diodon* and *Chilomycterus*.

The external and internal morphology of extant and fossil jawbones in *Diodon* accessed with micro CT imaging shows the oblique arrangements of numerous dental sheets. The crushing surface batteries exhibit 9–24 oblique-parallel dental sheets (Figs 4.16, 6.1–6.6, 7.1–7.9 and 7.13–7.14, S3 File). In contrast, *Chilomycterus* exhibits only a few dental sheets; the crushing-surface batteries are composed of 2 to 4 superficial dental sheets arranged horizontally and 5 to 6 inner dental sheets (Figs 4.1–4.15, 5.1–5.4, 5.6–5.11, 7.10, 7.12, 7.14, 7.16 and 8, S2, S4–S7 Files). The uppermost dental sheets of the crushing surface could be missing or broken in the fossil specimens.

## Results

Systematic Paleontology

Tetraodontiformes Regan, 1929 [111]

Diodontidae Bonaparte 1835 [112]

*Chilomycterus* Brisout de Barneville 1846 [113]

†*Chilomycterus tyleri* n. sp. Aguilera, Carrillo-Briceño and Rodriguez

**Fig 9:**
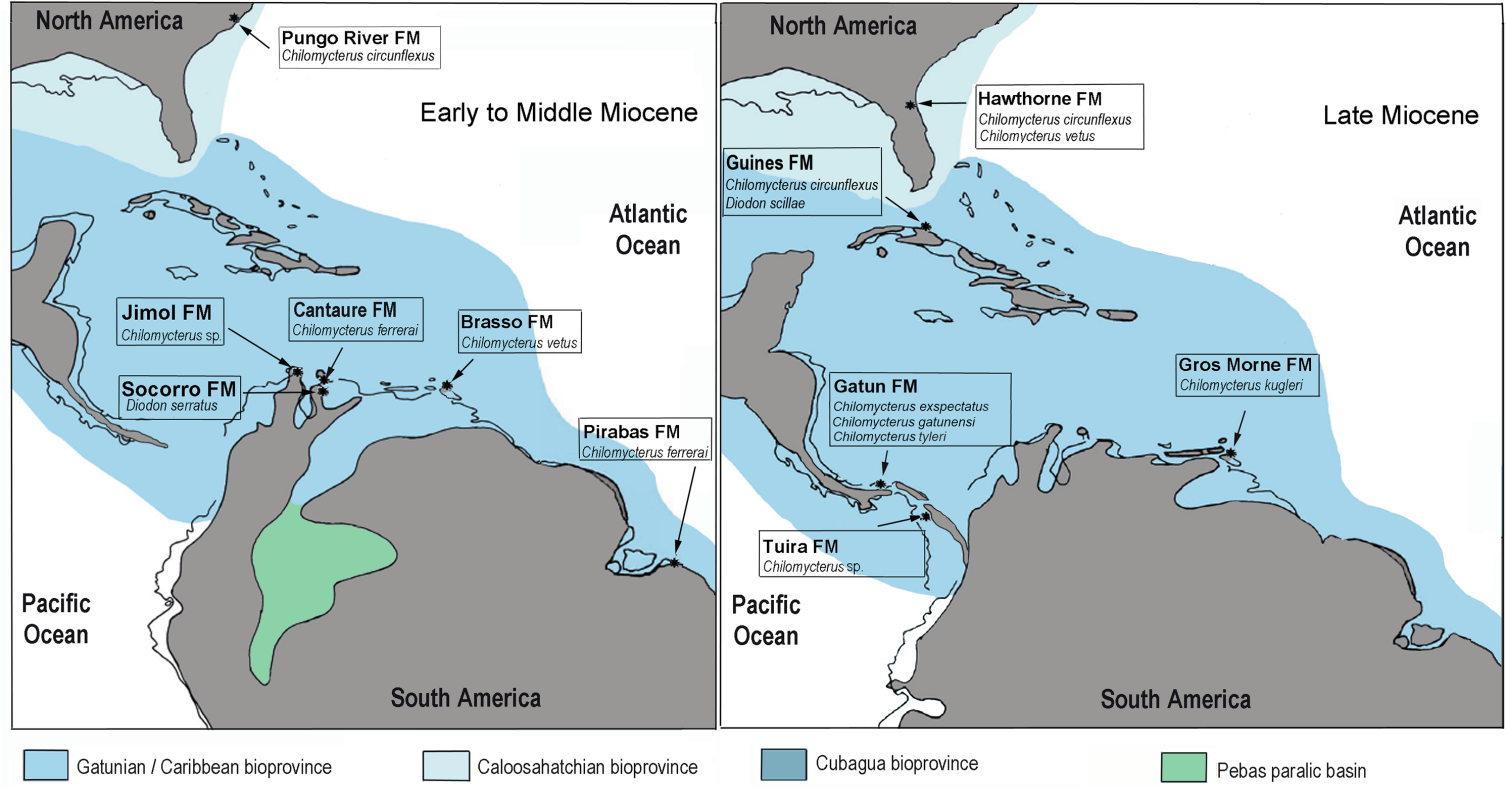
Paleogeographic range of Diodontidae. †*Chilomycterus circumflexus*, †*C*. *exspectatus* n. sp. † *C*. *ferrerai*, †*C*. *gatunensi*, †*C*. *tyleri* n. sp., †*C*. *kugleri*, †*C*. *vetus*, † *Diodon serratus* n. sp. and †*D*. *scillae*, from the Neogene Marine Tropical America. Modified schematic reconstruction [20,21,56,95,114,115,116,117,118].

**Figs** 4.1–4.3 and 9, S2 File

**Etymology.** In honor of James C. Tyler (Smithsonian Institution, Washington) in recognition of his many contributions to the knowledge of Tetraodontiformes.

**Holotype.** A single well-preserved specimen described (NMB P1208). The nearly complete lower jaw measures 99.8 mm in length (maximal preserved length) and 29.5 mm in width. The specimen is housed at the Naturhistorisches Museum of Basel (NMB), Switzerland.

**Type, locality, horizon and age.** Las Lomas, San Judas Tadeo, 9° 22' 58" N, 79° 49' 17" W, lower Gatun Formation, late Miocene, 11–10.5 Ma [54], Panama (Figs 1.1 and 2).

**Diagnosis.** †*Chilomycterus tyleri* n. sp. is distinguished from its modern and fossil conspecific of the genus *Chilomycterus* by having the grinding surface of the lower jaw arranged in five pairs of oval dental sheets in a flat to slightly depressed crushing surface; the frontal incisor tile-like small and in compact rows; the front edge of the fused jaws and the posterior margin are gently arched; the rear edge of the fused jaws is almost straight.

**Description.** Lower jaw thick, wide and long, fused to the opposite bone in the middle. The front edge of the fused jaws is gently arched; the proximal enlargement of both dentaries forms two robust and divergent branches with a deep depression in the internal angle. No large foramen can be observed. The mouth gap is moderate to large, even though the jaws may form a massive beak. The teeth are not protruding and are incorporated into the matrix of the beak-like jawbone. The frontal teeth are small incisor tile-like, arranged in successive and compacted rows. The internal series of teeth in the occlusal surface form a large horizontal tooth-plate battery for grinding. This crushing plate is divided into right and left halves and the two batteries are fused in the medial region. These tooth-plates are arranged in successive and internal stacked sheets in twenty-one flattened dental sheets. The occlusal surface is flat and slightly depressed. The horizontal plane of the crushing surface has four large dental sheets. A shallow groove separates the marginal massive incisor-like teeth and the occlusal crushing surface. Bellow the crushing battery and bone area a deep and wide ‘chamber’ is present in the rear of the jaw for muscle insertion (adductor mandibulae complex A2α, A2ß). Internally the bone structure reveals large and small channels for blood vessels and nerves. The maximal preserved length is 99.8 mm and width is 29.5 mm; the tooth plate crushing surface is 43.6 mm long and 17.3 mm width; the posterior chamber is 43.5 mm long and 9.4 mm high.

†*Chilomycterus ferrerai* (Santos and Travassos 1960) [29]

1960 †*Diodon ferrerai* Santos and Travassos: Pl. 3. Figs 11–13.

**Figs** 4.4–4.11 and 9.

**Material.** MN 2649-V (holotype), an upper jaw fragment, measures 16.2 mm in length (maximal preserved length) and 12.1 mm in width, Praia do Castelo, Ilha de Fortaleza, São João de Pirabas, Pará state, Brazil; MN 2637-V (paratype), a lower tooth plate battery, measures 16.8 mm in length and 10.6 mm in width, Ilha de Fortaleza, São João de Pirabas, Pará state, Brazil; MPEG 2084-V, a upper jaw, measures 24 mm in length and 10.9 mm in width, Praia de Atalaia, Salinópolis, Pará state, Brazil; DG UFRJ 305-P, a lower jaw fragment, measures 53 mm in length and 27 mm in width, Ilha de Fortaleza, São João de Pirabas, Pará state, Brazil; MPEG 1531-V, a lower tooth plate battery, measures 29.83 mm in length and 12.56 mm in width, Capanema, Mine B-17, Pará state, Brazil; MPEG 1775-V, a upper jaw fragment, measures 17.57 mm in length and 8.60 mm in width, Praia de Atalaia, Pará state, early Miocene Pirabas Formation, Brazil; UNEFM-PF-270, a upper jaw fragment measures 25.0 mm in length (maximal preserved length) and 15.0 mm in width, San José de Cocodite, Paraguaná Peninsula, Cantaure Formation, early Miocene, Venezuela.

**Description.** Upper jaw thick and wide fused to the opposite bone in the middle. The front edge of the fused jaws is strongly arched. The mouth gap is small, even though the jaws may form a massive beak. The teeth are not protruding and are incorporated in the matrix of the beak-like jawbone. The frontal teeth are distinctive, small; incisor tile-like, which fits with others; teeth and are arranged in successive and compact rows. The internal series of teeth in the occlusal surface form pairs of ovoid horizontal tooth-plates for grinding. These tooth-plates are divided into right and left halves and the two batteries are fused in the medial region. They are arranged in successive and internal stacked sheets into four flattened dental sheets. The occlusal surface is flat and shows the first three and eventually the fourth dental sheets of the horizontal series. A deep groove separates the marginal massive incisor-like teeth from the occlusal crushing surface.

The tooth plate battery of the lower jaw has a sub-rectangular or pear-shape. The tooth-plates are arranged in six to seven horizontal and successive sheets. The first two sheets are wide, sigmoid-shaped and clearly eroded from grinding. The five preserved dental sheets are visible in the rear edge of the tooth plate.

†*Chilomycterus exspectatus* n. sp. Aguilera, Carrillo-Briceño and Rodriguez

**Figs** 4.12–4.13 and 9

**Etymology.** The species name *exspectatus* refers to the turnover of the crushing dental plate adaptation depicted by this species during the clade's evolution.

**Type.** The holotype (NMB P1205) is a nearly complete upper jaw measuring 20.2 mm in length (maximal preserved length) and 9.45 mm in width. The paratype (NMB P1206) is a tooth battery of a lower jaw measuring 16.64 mm in length (maximal preserved length) and 9.12 mm in width. The specimen is housed at the Natural History Museum of Basel, Switzerland.

**Type locality, horizon and age.** Las Lomas (San Judas Tadeo), SJ14-1-1, lower Gatun Formation, late Miocene, 11–10.5 Ma [54], Panama (Figs 1 and 2).

**Diagnosis.** †*Chilomycterus exspectatus* n. sp. is distinguished from its modern and fossil conspecific of the genus *Chilomycterus* by having the upper tooth plate battery strongly arched, with two flattened occlusal dental sheets, followed by four dental sheets arranged in an oblique series with acute cutting edges. The lower tooth-plate battery is characterized by having a unique enlarged horizontal dental sheet in the occlusal surface.

**Description.** The upper tooth-plate battery is divided into right and left halves fused in the medial region, and arranged in successive and internal stacked sheets into six flattened dental sheets. The occlusal surface is flat and shows the two first arched dental sheets of the horizontal series. The rear four dental sheets are arranged in an oblique series forming individual grater-shaped cutting crests. The lower tooth-plate battery is ovoid in shape and characterized by having a unique enlarged horizontal dental sheet in the occlusal surface, followed by six internal stacked sheets in a horizontal battery.

*Chilomycterus* sp.

**Figs** 4.14, 4.15 and 9.

**Material.** NMB P1207, a nearly complete tooth plate battery measures 28.8 mm in length (maximal preserved length) and 17.4 mm in width. The specimen was collected in a small tributary of Rio Icuanati, Chucunaque-Tuira basin, Darien (PPP 1593), Tuira Formation (late Miocene, Tortonian), Panama. MUN-STRI-41506, a tooth plate battery measuring 13.0 mm in length (maximal preserved length) and 8.0 mm in width, early Miocene Jimol Formation, locality 290468, La Guajira Peninsula, Colombia.

**Description.** A fragmented tooth-plate battery, ovoid in shape and characterized by having two to three enlarged horizontal dental sheets in the occlusal surface, followed by almost seven internal stacked sheets in a horizontal battery.

†*Diodon serratus* n. sp. Aguilera, Carrillo-Briceño and Rodriguez

**Fig**s 4.16 and 9.

**Etymology.** The species name *serratus* refers to the narrow and serrated edge of the crushing dental sheet in the occlusal surface.

**Type.** The holotype AMU-CURS-760, is a tooth plate battery measuring 18.0 mm in length (maximal preserved length) and 11.0 mm in width. The specimen is housed at the Urumaco Museum of Paleontology, Venezuela.

**Type locality, horizon and age.** Coquinoid layer of the Cerro Alto, Quebrada Honda, Cerro Alto, 11° 12 ' 30''N, 70° 08' 12'' W, Socorro Formation, middle Miocene, Venezuela (Figs 1 and 2).

**Diagnosis.** †*Diodon serratus* n. sp. is distinguished from its modern and fossil conspecific of the genus *Diodon* by having distinctive flat, narrow and serrated sheet teeth in the occlusal surface of the plate battery.

**Description.** The tooth-plate battery is divided into right and left halves fused in the medial region, and arranged in a series of nine narrow, flattened and serrated tooth sheets. The occlusal surface is flat and shows the first expanded tooth sheet followed by eight series of flat tooth sheets with the anterior edge serrate, arranged like leaf forms.

†*Chilomycterus gatunensis* (Toula 1909) [31]

**Fig**s 5.9–5.11 and 9

**Material.** NHMW 1933/XVIII/167, holotype, upper jaw, late Miocene Gatun Formation, Panama.

**Remarks.** “Original description in Toula 1909” [31]

†*Chilomycterus circunflexus* (Leriche 1942) [34]

**Fig**s 5.6–5.8 and 9

**Material**: MNHNH-P2083, an upper jaw, probably late Miocene, La Cueva sin Nombre, La Havana; PIMUZ A/I 3651, a lower tooth plate battery, middle Miocene, Caspersen Beach 2, Venice, Florida, USA; AMNH19552, a tooth battery, Miocene, La Havana Province, Cuba.

**Remarks.** “Original description in Leriche 1942” [34]

†*Chilomycterus kugleri* (Casier 1958) [33]

**Figs** 5.1–5.3 and 9

**Material**: NMB-Ant.58 (old fossil catalog number), holotype, tooth plate battery, late Miocene, Gross Morne Formation, Trinidad.

**Remarks.** “Original description in Casier 1958” [33]

†*Chilomycterus vetus* (Leidy 1877) [37]

**Figs** 5.4–5.5 and 9

**Material**: NMB-Ant. 57 (old fossil catalog number), upper jaw, middle Miocene Tamana Formation, Trinidad.

**Remarks.** “Original description in Leidy 1877” [37]

†*Diodon scillae* Agassiz 1843 [61]

**Figs** 6.5–6.6 and 9

**Material**: MNHNH- P3646, tooth plate battery, Cueva sin Nombre, La Havana, Cuba; FF8030 (= AMNH8030), tooth plate battery, La Havana, Cuba; FF14448 (= AMNH14448), tooth plate battery, unknown locality.

**Remarks.** “Original description in Agassiz 1843” [61]

## Discussion

Diodontidae fossil jawbones and tooth plate battery preserved in the sedimentary basins from the early to late Miocene Tropical Western Central Atlantic (TCWA) and from the Miocene Tropical Eastern Central Pacific (TECP), reveal a valuable opportunity to understand the paleobiogeography of Diodontidae fauna and contribute to elucidate the macroevolutionary responses in coastal faunule affected by the paleoceanographic and paleoenvironmental changes in the region caused by the tectonic dynamics and finally the severance of the Central American Seaway by the uplift of the Panamanian isthmus, the complete Atlantic-Pacific oceans isolation and the final configuration of the Caribbean Sea (Fig 9). These geological scenarios are needed to fully understand under regional researches in response of the continuous debate [3,18,19] and the expectation of future paleontological research.

**Fig 10:**
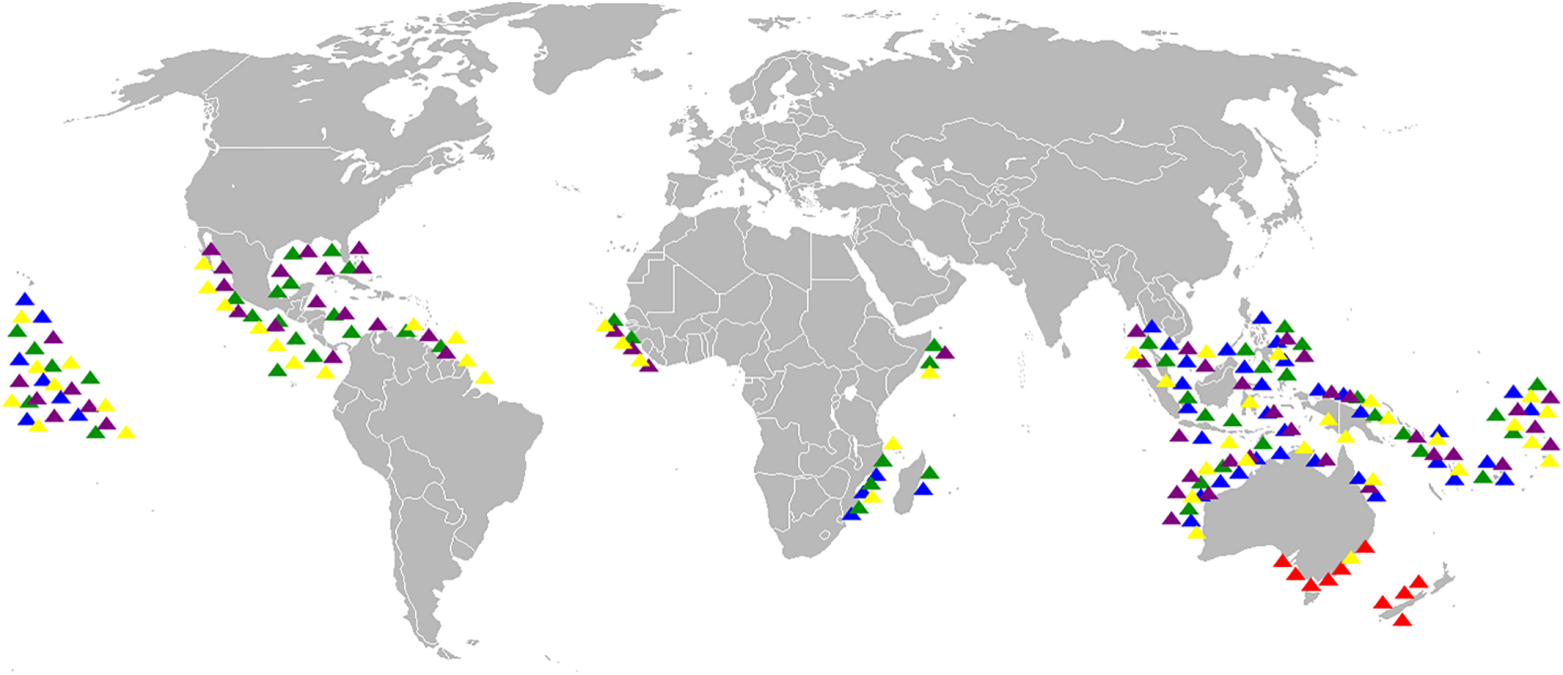
Biogeographic distribution of Recent *Diodon* species. *D*. *eydouxii* (yellow triangle), *D*. *liturosus* (blue triangle), *D*. *nicthemerus* (red triangle), *D*. *holocanthus* (purple triangle) and *D*. *hystrix* (green triangle). Modified from Froese and Pauly [67], Robertson and Allen [68] and Robertson and Tassell [69].

Fossil *Diodon* is present in both the Atlantic and Pacific oceans, whereas the distribution of *Chilomycterus* is highly asymmetrical, with only one species in the Pacific. *Diodon* appears to have been as abundant in the Caribbean/Western Atlantic during the Miocene as it is there today (Fig 10).

**Fig 11:**
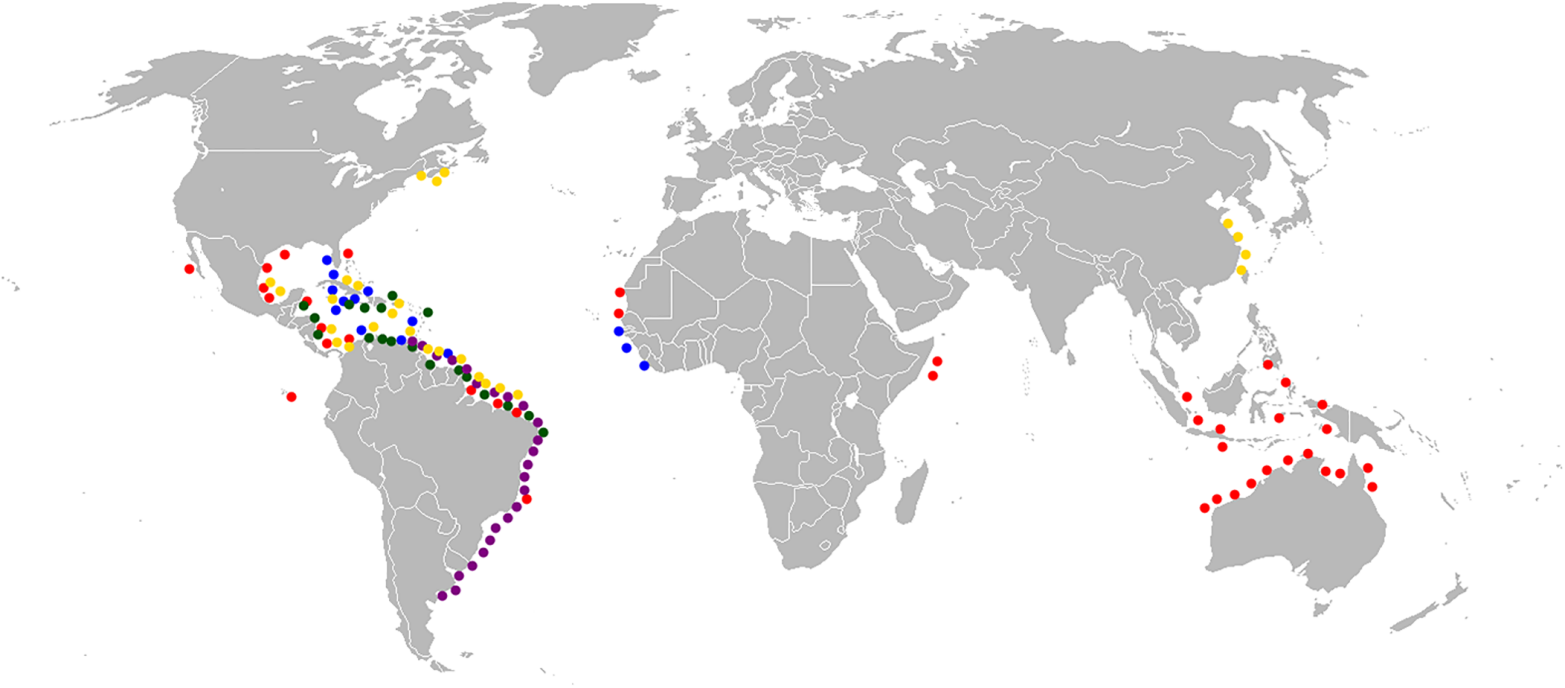
Biogeographic distribution of Recent *Chilomycterus* species. *C*. *antennatus* (blue circle), *C*. *antillarum* (green circle), *C*. *reticulatus* (red circle), *C*. *schoepfii* (yellow circle) and *C*. *spinosus* (purple circle). Modified from Froese and Pauly [67], Robertson and Allen [68] and Robertson and Tassell [69].

Amphi-American fossil marine assemblages, relict species, paciphile or germinate species records have been treated at length for corals, crustaceans and fishes [20,21,22,23,117,119,120]. The occurrence of a *Chilomycterus* species in the late Miocene of a Pacific deposit (Tuira FM) requires more research as it is a pattern opposite to the more traditional paciphile pattern from the Proto-Caribbean.The widely circumtropical distribution of Diodontidae (Figs 10 and 11) and the available data suggests that the diversification of Neogene amphi-American species started during the early Miocene, as phylogenies have suggested [49,51,52]. However, unavailable Eocene-Oligocene units in the study area with associated Diodontidae fossil fauna (Fig 3) or specimens from museum collections from Tropical America are the limiting to corroborate older divergence (i.e. compare with fossil records from the Eocene of Monte Bolca, Italy [121], Northern Caucasus, Russia [122]).

The Diodontidae are durophagous fishes that use the power of the fused jaws and the crushing tooth battery to feed on prey with a hard protected shell (mollusks), carapace (crustaceans) or test (echinoids) [41,42,43,44]. Consequently the broad distribution in tropical marine shallow waters over sandy, rocky and coral reef bottoms is likely to be related to the available food resources in coastal paleoenvironments, particularly the high abundance and diversity of Neogene tropical American mollusks [54,72,89,99,123], crustaceans [12,124,125,126] and echinoids [127,128,129,130] in all of sedimentary basins where the fossils were found.

Stratigraphical and fasciological contextual interpretation of the formations treated here (Fig 2) reveal that fossil fish assemblage where the Diodontidae specimens came inhabit over sandy and lime bottom in shallow water and were characterized by the presence of demersal teleostean fish [12, 23, 55, 57, 101,131] mostly Sciaenidae, some of then associated with early estuarine system in the Proto-Caribbean, or bathypelagic species mostly Mycthophidae [21] with diurnal/nocturnal displacement in the water column associated with high planktonic productivity. The elasmobranch fauna are represented mostly by Carcharhinidae [104, 132] that inhabit coastal shallow water.

The similarity of the marine fauna preserved in Miocene sediments from Ecuador, Colombia, Costa Rica, Panama and Venezuela promoted the designation of the Gatunian Faunal Province (between TCWA and TECP), named after the late Miocene Gatun Formation in Panama [117,133,134,135,136], whereas the fauna is sufficiently distinct post-isthmus closure to warrant the use of the term Pleistocene Caribbean Province [117]. Early Miocene fish assemblages to further characterize the Brazilian equatorial fish faunas amongst these Provinces has yet to be explored [137,138], and reflect the lithostratigraphic sequences across the Proto-Caribbean and the shift from one to the other broadly records a widespread biological extinction and turnover in the TCWA and TECP marine fauna.

We believe that the diodontid record presented here represents a baseline for future research, as ongoing paleontological research in the American tropics continues to fill the gaps in the Neogene record.

## Supporting information

S1 File Examined specimens. (DOC)

S2 File Micro CT reconstruction of fossil jaw from †*Chilomycterus tyleri* n. sp. NMB P1208. (AVI)

S3 File Micro CT reconstruction of jaw from the extant species *Diodon hystrix* UFF ZO426. (AVI)

S4 File Micro CT reconstruction of jaw from the extant species *Chilomycterus spinosus* UFF ZO313. (AVI)

S5 File Micro CT reconstruction of jaw from the extant species *Chilomycterus spinosus*. UFF ZO314, 92 mm total body length, 72 mm standard length.
(MOV)

S6 File Micro CT reconstruction of jaw from the extant species *Chilomycterus spinosus*. UFF ZO312, 159 mm total body length, 120 mm standard length.
(MOV)

S7 File Micro CT reconstruction of jaw from the extant species *Chilomycterus spinosus*. UFF ZO315, 276 mm total body length, 230 mm standard length.
(MOV)

S1 Fig Number of dental sheets in *Diodon* (circle) and *Chilomycterus* (triangle), as a function of the body size (standard length). Blue color: referential data from Tyler [91]; green color: Micro CT data from the present work.
(TIF)
