# Supplementary material for: Neogene Proto-Caribbean porcupinefishes (Diodontidae)
Source: PLoS One. 2017 Jul 26;12(7):e0181670. doi: 10.1371/journal.pone.0181670 (PMC5528887; doi:10.1371/journal.pone.0181670)
Supplement: S1 File — (DOC) [file pone.0181670.s001.doc]

**S1 Appendix.** Examined specimens

**Fossil**

†*Chilomycterus circunflexus* MNHNH-P2083, PIMUZ A/I 3651.

† *Chilomycterus exspectatum* n. sp*.*, MNB P1205, MNB P1206.

†*Chilomycterus ferreirai* MPEG 2084-V, MN 2649-V, UNEFM-PF-270.

†*Chilomycterus gatunensis* NHMW 1933/XVIII/167.

†*Chilomycterus kugleri* NMB-Ant.58.

†*Chilomycterus tyleri* n. sp. NMB P1208.

†*Chilomycterus vetus* NMB-Ant.57.

*Chilomycterus* sp. MNB P1207, MUN STRI- 41506.

† *Diodon scillae* MNHNH- P3646.

†*Diodon sigma* MNB-T.A.121.

†*Diodon serratus* n. sp., AMU-CURS-760.

**Extant**

*Diodon holocanthus* ANSP 102787, MUN STRI-43972.

*Diodon hystrix* ANSP 102789, ANSP 109515, MUN STRI-43973, UFF ZO426.

*Diodon liturosus* ANSP 109145.

*Chilomycterus antennatus* MUN STRI-43971.

*Chilomycterus antillarum* MNHN 971-9506.0023.

*Chilomycterus* *nicthemerus* AMNH 219858.

*Chilomycterus schoepfii* ANSP 109514.

*Chilomycterus spinosus* UFF ZO132, ZO312, ZO313, ZO314, ZO315.
