# Supplementary figures and images for: Neogene Proto-Caribbean porcupinefishes (Diodontidae)
Source: PLoS One. 2017 Jul 26;12(7):e0181670. doi: 10.1371/journal.pone.0181670 (PMC5528887; doi:10.1371/journal.pone.0181670)

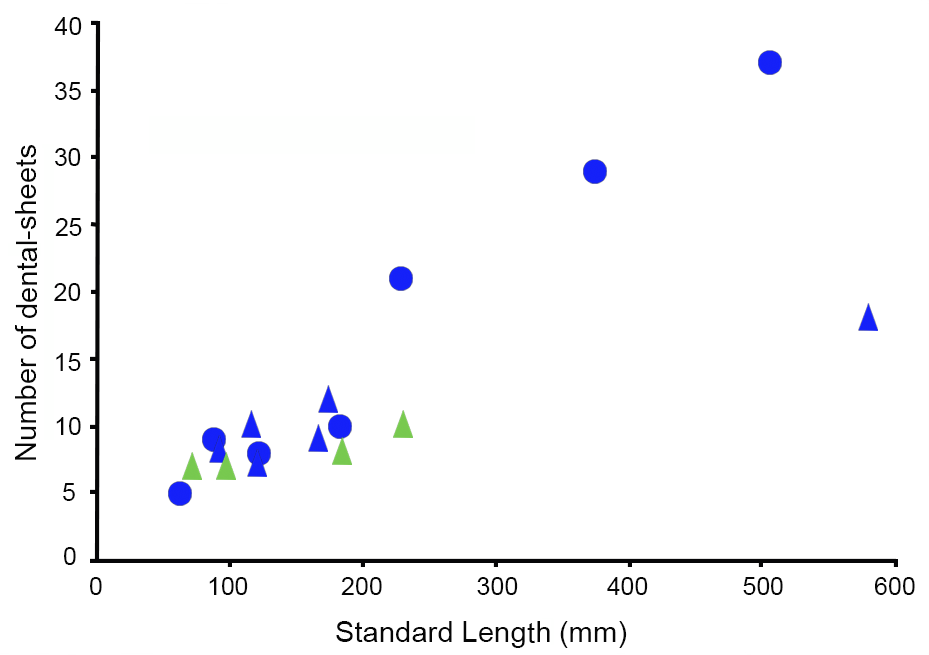

Supplement: S1 Fig — Blue color: referential data from Tyler [91]; green color: Micro CT data from the present work. (TIF) [file pone.0181670.s008.tif]
